# The “Wave Sign” as Evidence of a “GUCCI” (Gluteal Undersurface Concealed Crescent Injury) Lesion: A Case Report

**DOI:** 10.1155/cro/5547185

**Published:** 2026-02-19

**Authors:** Mark M. Kodsy, Jensen G. Kolaczko, Trevor Wait, Ognjen Stevanovic, James Genuario

**Affiliations:** ^1^ MetroHealth Medical Center, Cleveland, Ohio, USA, metrohealth.org; ^2^ University Hospitals Drusinsky Sports Medicine Institute, Beachwood, Ohio, USA; ^3^ Case Western Reserve University School of Medicine, Cleveland, Ohio, USA, case.edu; ^4^ UCHealth Steadman Hawkins Clinic Denver, Englewood, Colorado, USA; ^5^ University of Colorado School of Medicine, Aurora, Colorado, USA, ucdenver.edu

## Abstract

A 63‐year‐old female presented with left lateral hip pain consistent with greater trochanteric pain syndrome (GTPS) and magnetic resonance imaging (MRI) findings of high‐grade gluteal tendinosis and partial tearing. She failed conservative management and was thus indicated for endoscopic greater trochanteric bursectomy and gluteal tendon repair. Intraoperatively, the superficial gluteus medius tendon fibers were intact, but a “wave sign” was present on dynamic examination. Subsequent investigation revealed a proposed GUCCI (gluteal undersurface concealed crescent injury) lesion, which underwent endoscopic repair. This case highlights the importance of dynamic endoscopic examination of the gluteal tendons, as identification of a trochanteric “wave sign” may reveal clinically significant pathology concealed beneath intact bursal‐sided fibers.

## 1. Introduction

Greater trochanteric pain syndrome (GTPS) is a multifactorial condition characterized by lateral hip pain arising from pathology involving the peritrochanteric soft tissues. While repetitive microtrauma and friction between the iliotibial band (ITB) and the greater trochanter have historically been implicated, contemporary evidence suggests that GTPS is additionally a tendon‐driven pathology involving gluteal tendinopathy or tearing, often with concomitant trochanteric bursitis [1–4]. The trochanteric bursa serves a protective role in reducing friction, and pain does not arise from friction alone but rather from disruption of normal hip mechanics, altered muscle cocontraction, and tendon degeneration. GTPS accounts for up to 20% of painful hip evaluations and predominantly affects females over 40 years of age at a reported ratio of approximately 4:1. This disproportion is multifactorial, related to pelvic morphology and anatomy, as well as functional biomechanical factors such as gluteus medius weakness or altered activation relative to the ITB during gait, which can further exacerbate lateral hip loading [3, 5, 6].

Patients with GTPS report lateral peritrochanteric pain with lying on the affected side and activities with hip abduction [4]. Physical exam will reveal tenderness to palpation over the greater trochanter, weakness/pain with resisted hip abduction, and possibly a Trendelenburg gait/sign [4, 7]. The treatment for GTPS consists initially of conservative measures such as physical therapy, anti‐inflammatories, and injections (corticosteroids or platelet‐rich plasma [PRP]) with a success rate over 90%^14^. For refractory cases, surgical interventions exist in the form of greater trochanteric bursectomy, ITB release, and gluteal tendon repair [1, 4, 5]. Surgery for GTPS can be done using an open approach or endoscopically. Both techniques demonstrate similar improvements in functional outcomes with good to excellent results, reduced pain, improved abductor strength, and similar retear rates [8, 9]. Endoscopic techniques, however, have the benefit of fewer complications [8, 9]. Endoscopic techniques may also allow better dynamic evaluation of partial‐thickness tears and identification of an intraoperative “wave sign.”

## 2. Statement of Informed Consent

This case report qualifies for an exemption for informed consent as it does not present more than minimal risk and all the research procedures fit within the exemption categories in the federal IRB regulation. Therefore, due to the nature of this case study, no IRB review is necessary. The information presented in this case study is not generalizable and only applies to one subject/patient, and informed consent from the individual patient in this study was obtained in accordance with institutional policy. Please contact COMIRB at comirb@ucdenver.edu or 303‐724‐1055 with questions/concerns.

## 3. Case Report

**Figure 1:**
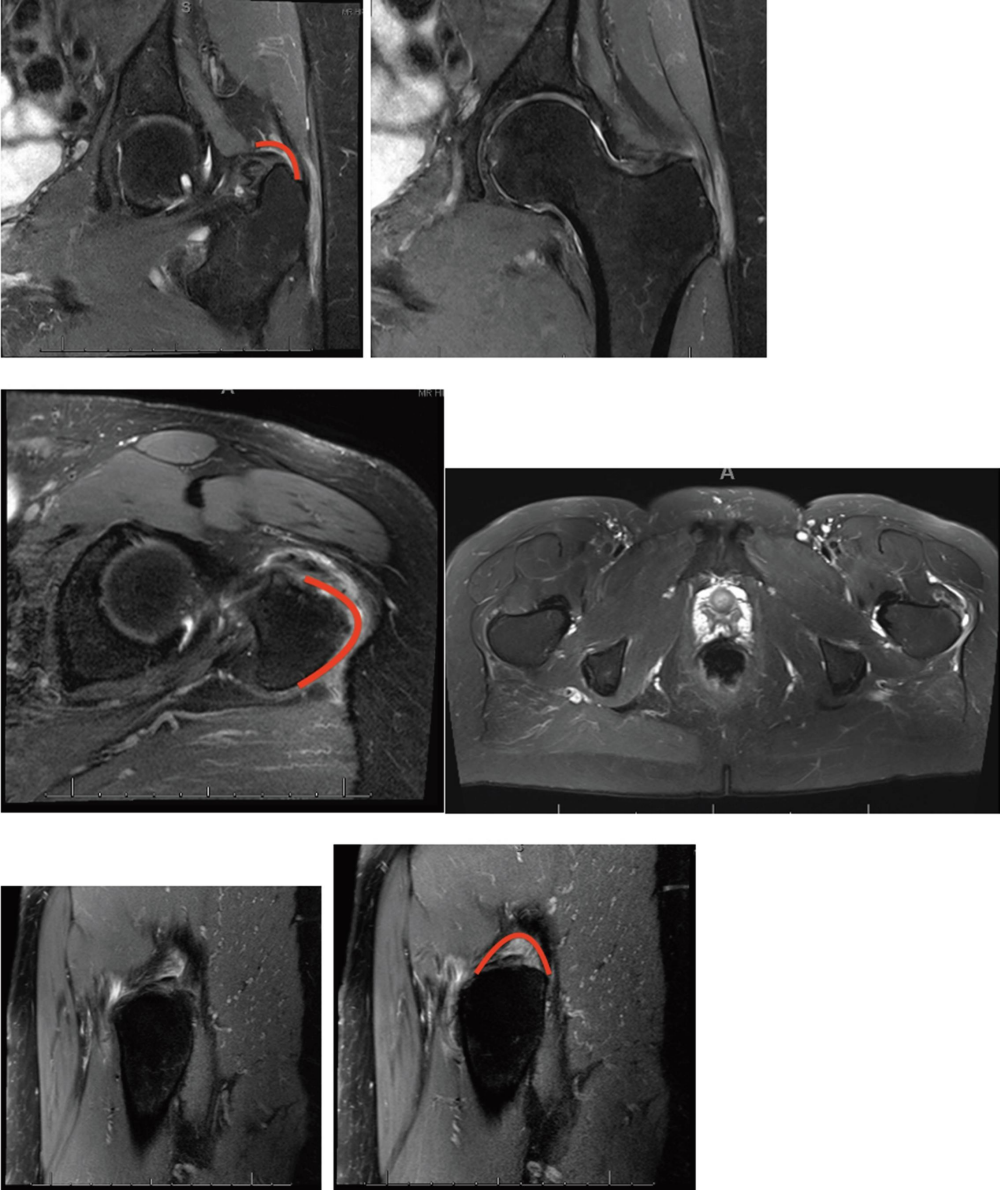
T2 coronal, axial, and sagittal 3‐T magnetic resonance imaging (MRI) illustrating left‐sided high‐grade tendinosis and partial tearing of the left gluteus medius. The red line demonstrates crescent shape tearing of the gluteus medius and minimus tendons (GUCCI lesion).

This is a 63‐year‐old female with chief complaint of 2 years of ongoing left lateral–sided hip pain. She underwent extensive nonoperative treatment including activity modification, nonsteroidal anti‐inflammatory medications, focused physical therapy emphasizing hip abductor strengthening, multiple corticosteroid injections, and leukocyte‐rich PRP injections, without sustained symptomatic relief. Her physical exam consistently revealed point tenderness to palpation over the posterior aspect of the greater trochanter. She initially had 5/5 hip abduction strength on manual muscle testing, though this was associated with lateral hip pain. Over time, despite nonoperative management, she developed pain‐limited weakness with resisted hip abduction, consistent with progressive abductor dysfunction. Advanced imaging was obtained to further evaluate her pathology. Magnetic resonance imaging (MRI) revealed progressive high‐grade tendinosis and partial tearing of the left gluteus medius and minimus. We define this pathology as a proposed gluteal undersurface concealed crescent injury (GUCCI), describing a crescent‐shaped undersurface tear of the gluteus medius and/or minimus beneath intact bursal‐sided tendon fibers (Figure 1).

**Figure 2:**
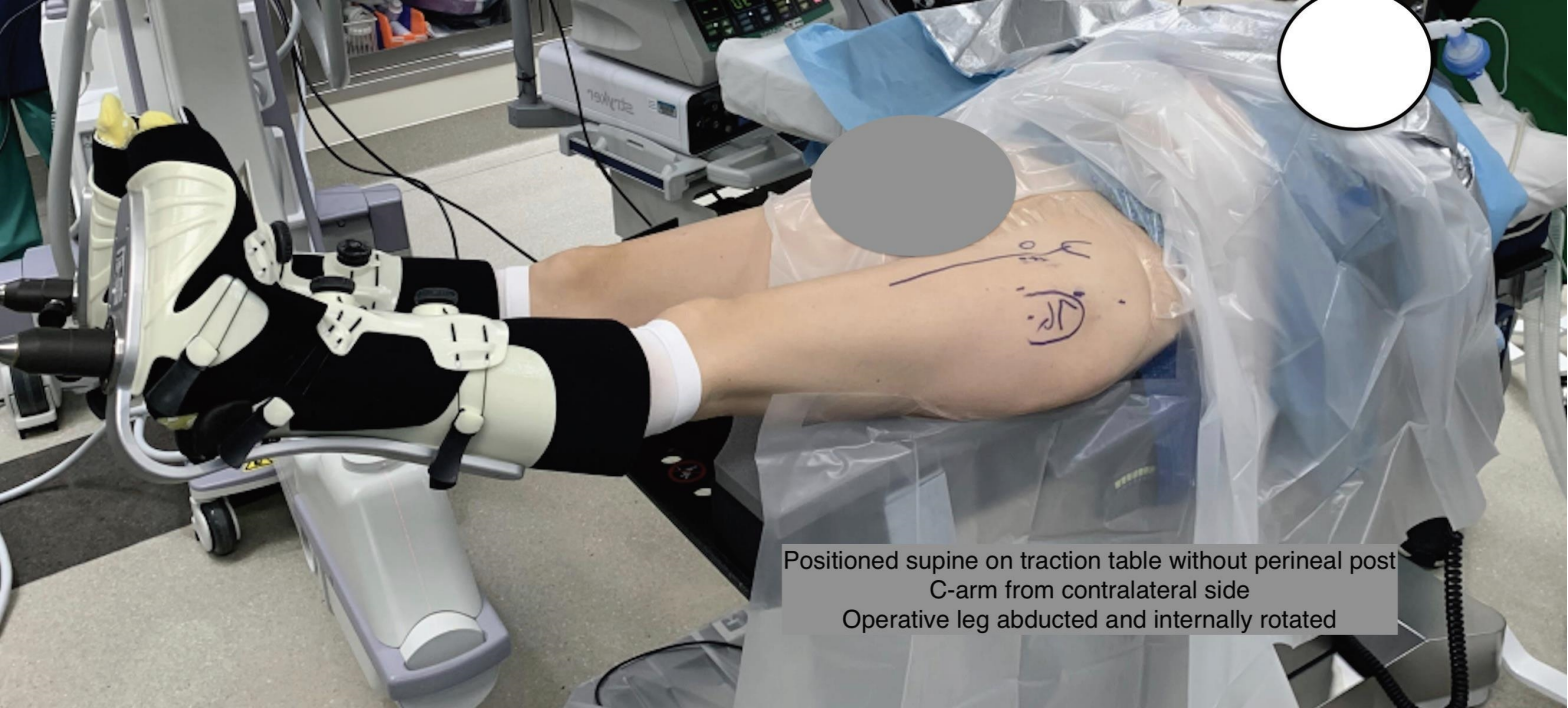
Intraoperative patient positioning. The patient is placed supine. The operative extremity is abducted and internally rotated. The bed is then “airplaned” away from the surgeon by 20°.

**Figure 4:**
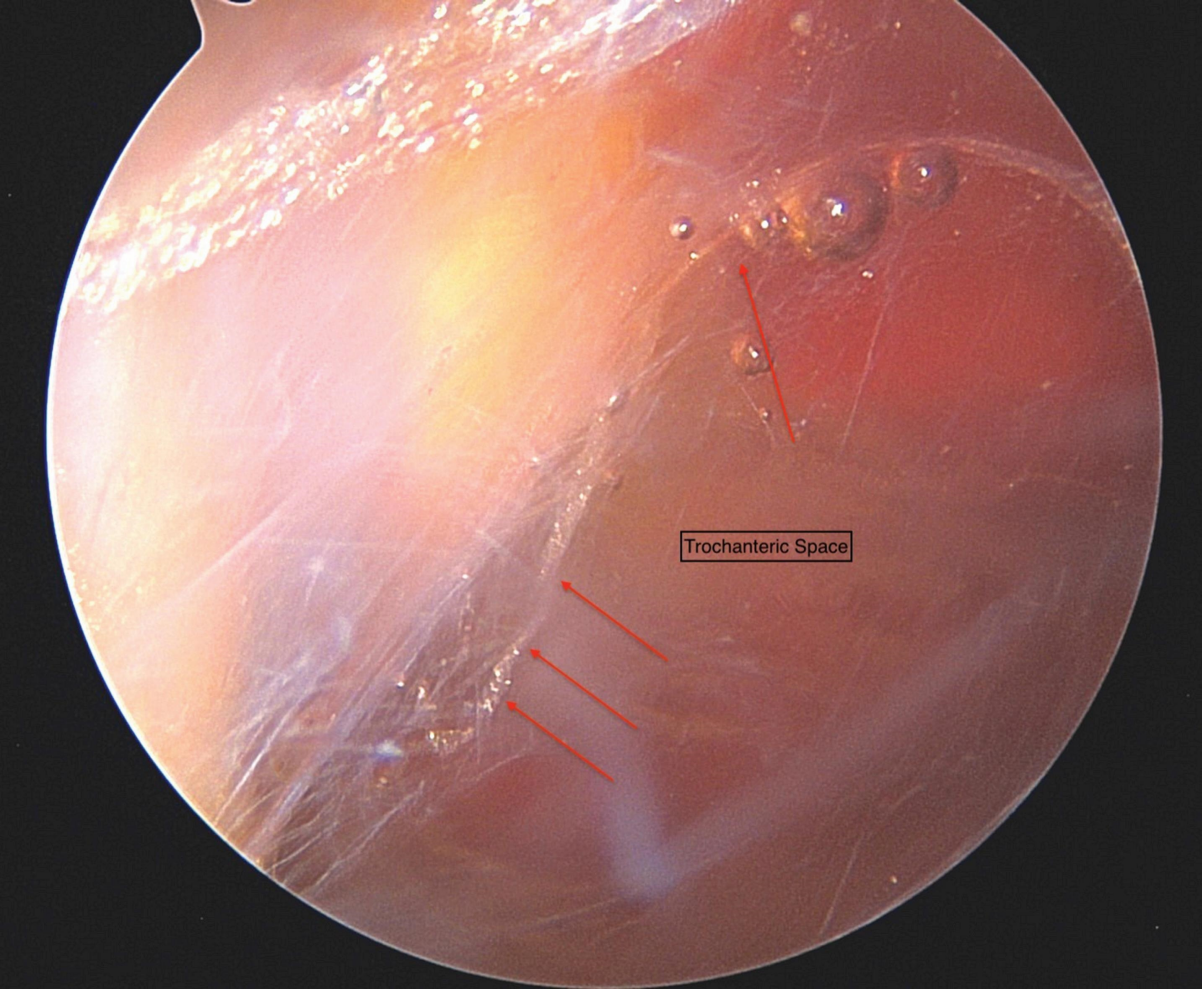
Endoscopic images from distal gluteal viewing portal and red arrows showing extensive bursitis in the trochanteric space.

**Figure 5:**
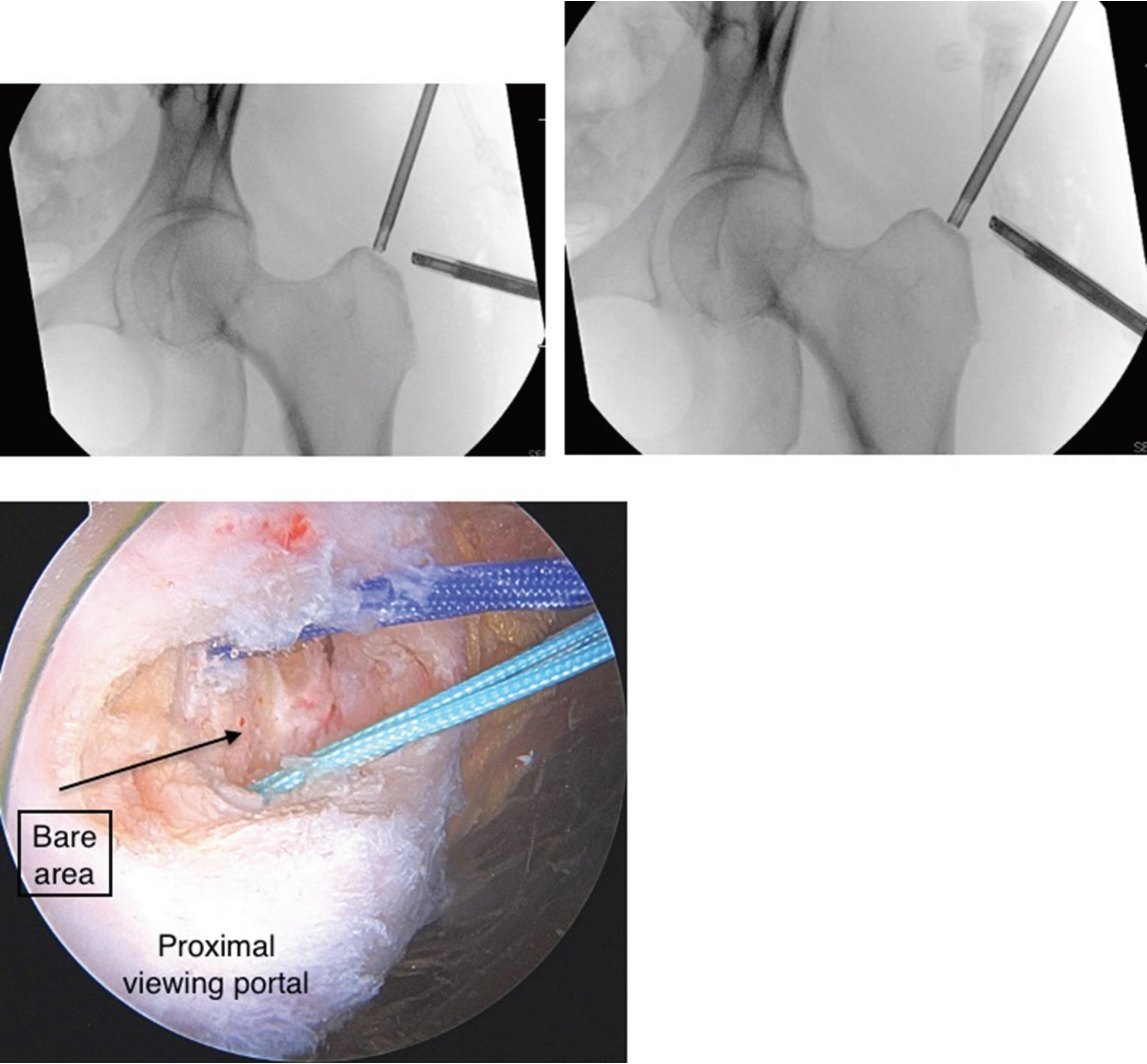
Intraoperative fluoroscopy and endoscopic images from proximal gluteal working portal which illustrate suture anchor placement at the junction of the anterior lateral facet and another anchor at the junction of the lateral and posterior facet.

**Figure 6:**
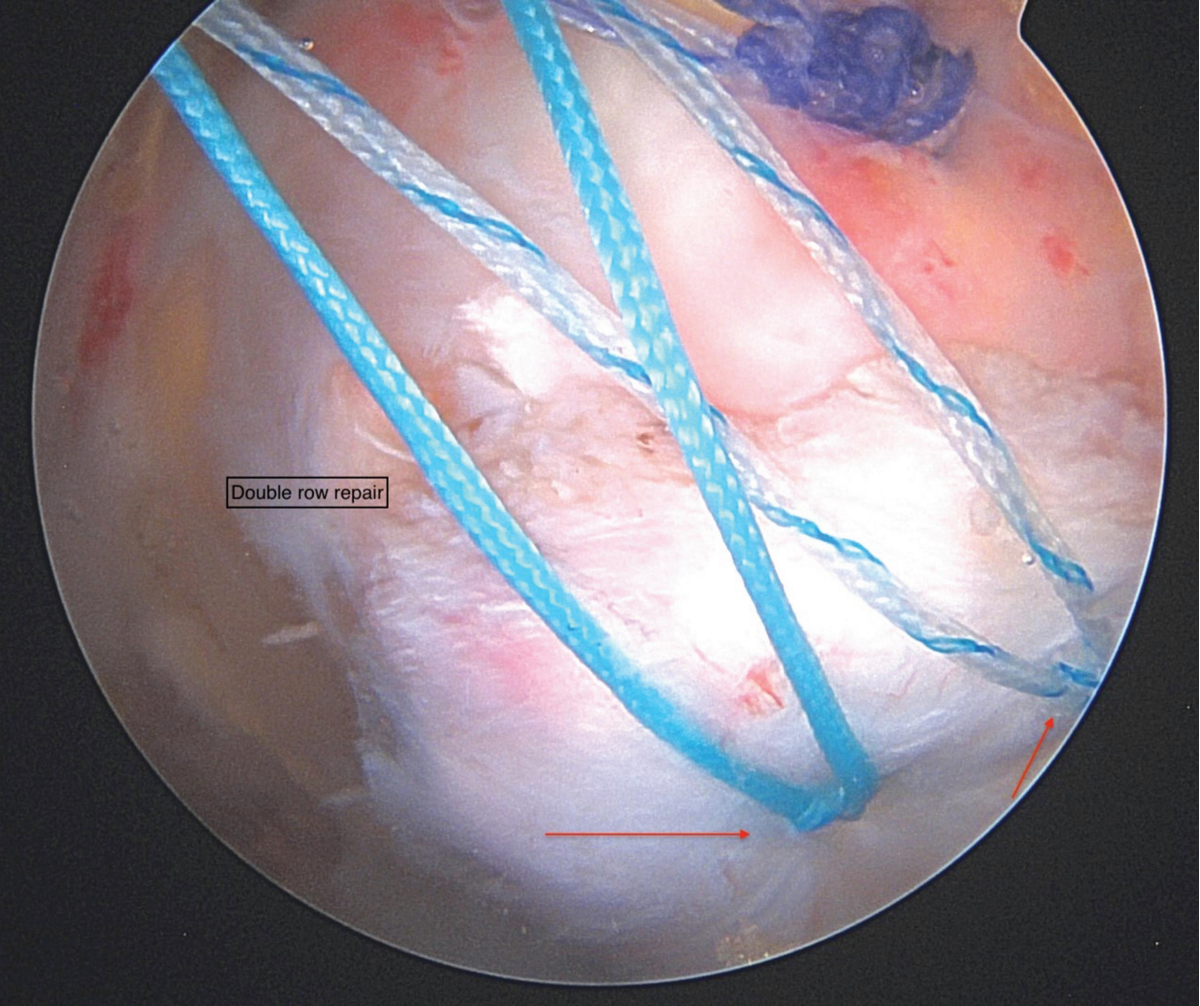
Endoscopic image from proximal the gluteal working portal and red arrows demonstrating suture limbs passed in a horizontal mattress fashion through the tendon and tied for the proximal row of the repair.

**Figure 7:**
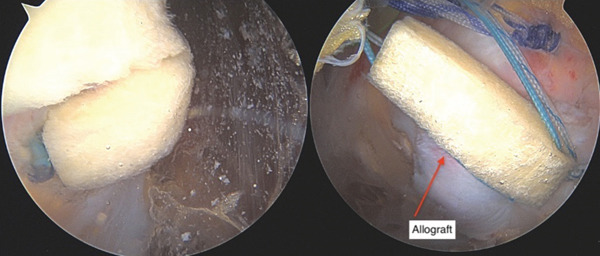
Final endoscopic images from proximal gluteal viewing portal demonstrating double row repair which was performed with the aid of dermal allograft augmentation due to poor tissue quality.

She exhausted all conservative measures and was thus indicated for surgical intervention. The patient was brought back to the operating theater where general anesthetic was administered and preoperative antibiotics were given. The patient was placed supine on the Stryker Guardian Table (Pivot Guardian, Stryker Endoscopy, Kalamazoo, Michigan) where all bony prominences were appropriately padded (Figure 2). The bed was air‐planed away, leg abducted, and internally rotated. The hip was prepped and draped in standard sterile fashion. Portal placement was established under fluoroscopic guidance: Three portals were used primarily for this technique and highlighted in Figure 3a,b. These included the proximal anterolateral (AL) portal, along with a distal gluteal viewing portal (VP) and proximal gluteal working portal (WP) made approximately 4–5 cm distal and proximal to the AL portal, respectively (Figure 3a,b). Diagnostic endoscopy was performed via the proximal AL VP, which showed extensive bursitis (Figure 4). An endoscopic trochanteric bursectomy was performed with a combination of a shaver and ablation wand through the distal gluteal VP. The superficial gluteal tendon fibers appeared intact; however, dynamic probing revealed a trochanteric “wave sign,” characterized by abnormal undulation of the intact bursal surface with motion (Video S1). A small longitudinal incision was made in line with the intact bursal‐sided gluteus medius fibers to allow direct visualization and assessment of the undersurface tendon pathology while preserving the superficial tendon layer. The proximal and lateral facets were bare with a deep undersurface tear of the medius and complete tear of the minimus consistent with a GUCCI lesion (Video S1). A bleeding bone bed was prepared using a burr. Two double‐loaded 2.3 mm all‐suture anchors (Iconix, Stryker, Kalamazoo, Michigan) were placed at the junctions of the AL and posterolateral facets (Figure 5). All eight limbs were passed through the tendon and tied to create the proximal row (Figure 6). A double‐row repair was selected to maximize footprint contact and fixation strength, particularly given the poor tissue quality and undersurface nature of the tear. Distal anchors were placed along the vastus ridge, and allograft augmentation (DERMIS ON DEMAND, Johnson & Johnson, New Brunswick, New Jersey) was utilized to reinforce the repair and enhance biologic healing potential (Figure 7). The hip was brought through range of motion (ROM) and the gluteal repair remained stable. All excess fluid was removed from the hip, and portals were closed with Monocryl (Ethicon, Johnson & Johnson, New Brunswick, New Jersey) and nylon sutures.

Figure 3(a) Skin marking of anatomic landmarks and portal position. ASIS: anterior superior iliac spine, AL: proximal anterolateral portal; MA: modified anterior portal; DALA: distal anterolateral portal; VP: viewing portal, WP: working portal. The yellow highlighted portals are utilized for bursectomy and gluteal tendon repair. (b) Intraoperative fluoroscopy illustrating access into the trochanteric space from a distal gluteal viewing portal.

**Figure:**
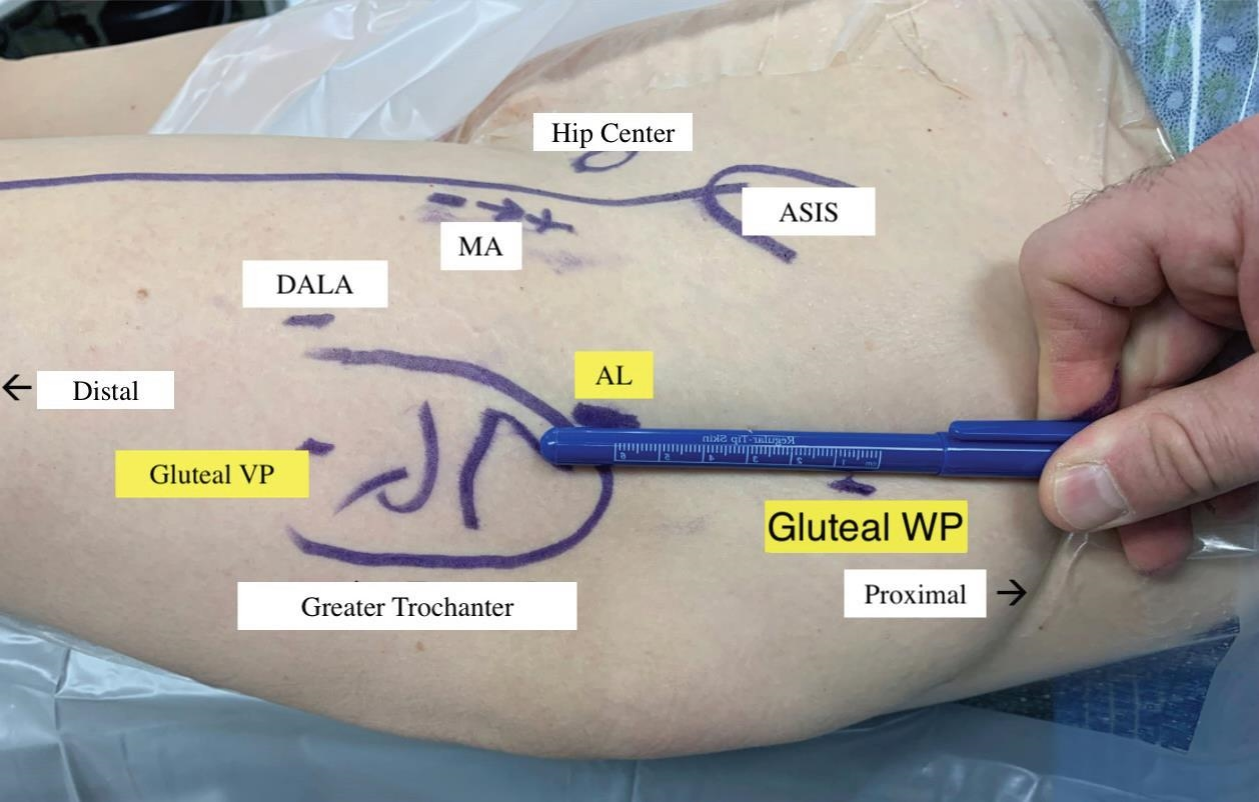
(a)

**Figure:**
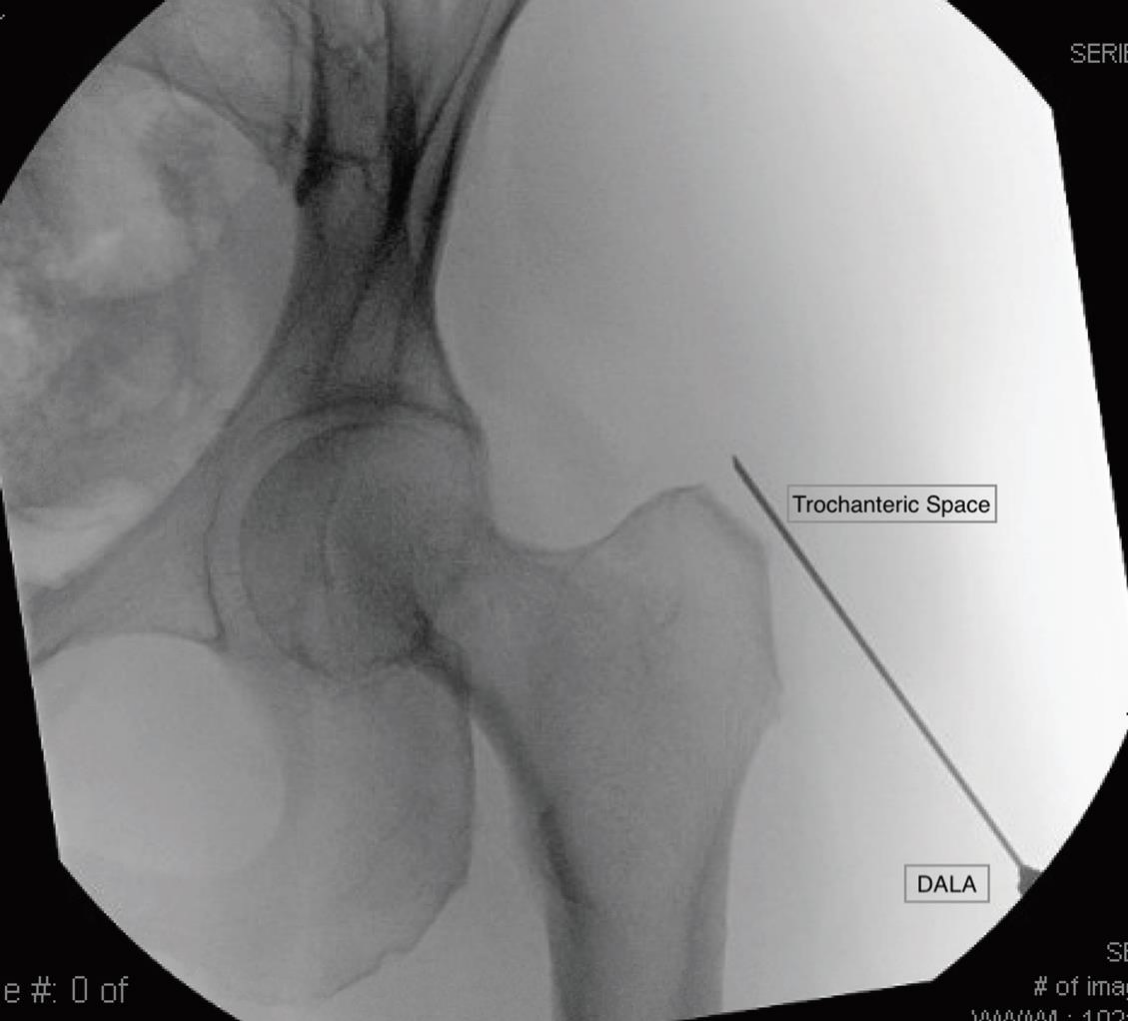
(b)

Postoperative rehabilitation involves a high‐grade partial‐thickness tear repair program instituted under the direction and supervision of a certified physical therapist. This rehabilitation protocol is a standardized, nonproprietary program based on previous generally published protocols for endoscopic gluteal tendon repair [10]. Phase 1 includes 4 weeks of touchdown weightbearing, and a hip abduction orthosis set at 30°–75° of hip flexion to mitigate tension on the repaired tendons. Phase 2 begins progression toward full weightbearing and off crutches in 1–2 weeks and working toward full ROM. Phase 3 focuses on restoration of normal gait, no Trendelenburg with single leg movements, and normal bilateral squat mechanics. Phase 4, the final phase, begins when the patient obtains full hip abduction and extension strength, symmetrical single leg mechanics, and full painless ROM. After this phase, the patient is allowed to return to full activity.

At 4 months postoperatively, the patient had near symmetric full painless ROM of her operative hip. She was able to ambulate with a nonantalgic gait. She demonstrated 4/5 hip abduction strength on manual muscle testing in the lateral decubitus position. She continued toward her end goal of regaining strength and returning to full activity.

## 4. Discussion

GTPS is a known cause of peritrochanteric hip pain which causes pain and dysfunction. Most patients will have symptom resolution with conservative management. However, for those with refractory symptoms, endoscopic surgical intervention is a reliable and effective option. This case highlights the importance of recognition of an intraoperative “wave sign,” which revealed a GUCCI lesion requiring repair.

Historically, GTPS was thought to be due to isolated bursitis. However, gluteal tendon pathology and trochanteric bursitis are concomitant findings in patients with GTPS. Gluteus medius tears represent the most common finding with complete tears in 46% and partial tears in 38% of cases [11]. Increasing attention has been directed toward recognition of gluteus minimus pathology. Analogous to partial articular supraspinatus tendon avulsion (PASTA) injuries in the shoulder, a GUCCI lesion represents an undersurface tear of the gluteus medius and/or minimus concealed beneath intact bursal‐sided fibers [5]. The majority of complete distal gluteus minimus tendon tears show continuity of distal tendon fibers with the proximal vastus lateralis, which tether and limit proximal tendon retraction [12]. Failure to recognize and address these concealed undersurface tears may lead to persistent abductor weakness, fatty degeneration, impaired tendon healing, and inferior functional outcomes, including suboptimal results following gluteus maximus transfer [5, 12].

This case underscores the importance of recognizing a trochanteric “wave sign” as a dynamic intraoperative indicator of concealed undersurface pathology. Endoscopic techniques facilitate identification and treatment of these lesions while offering outcomes comparable to open repair with fewer complications. Surgeons performing endoscopic bursectomy or gluteal tendon repair should maintain a high index of suspicion for GUCCI lesions when a wave sign is observed.

Potential complications of endoscopic gluteal tendon repair include persistent pain, incomplete healing, anchor failure, infection, and postoperative stiffness. Limitations of this report include its single‐patient design, short‐term follow‐up, and lack of validated patient‐reported outcome measures. Nevertheless, this case highlights an important diagnostic and therapeutic consideration that may significantly influence surgical decision‐making and patient outcomes.

## Funding

No funding was received for this manuscript.

## Conflicts of Interest

Author J.G. has IP royalties, is a paid consultant, is a paid speaker and presenter, and has research support from Stryker. These disclosures were not relevant to the current work.

## Supporting information


**Supporting Information** Additional supporting information can be found online in the Supporting Information section. Video S1: Endoscopic images depicting the “wave sign” which indicate an underlying GUCCI lesion. An endoscopic ablator is used to make a small superficial incision in line with the intact bursal side of the gluteus medius fibers. The proximal and lateral facets were found to be bare. There was a deep undersurface tear of the gluteus medius and a complete tear of the gluteus minimus consistent with a GUCCI lesion.

## Data Availability

Data sharing is not applicable to this article as no datasets were generated or analyzed during the current study.
